# Inflammatory Profile of Older Adults in Response to Physical Activity and Diet Supplementation: A Systematic Review

**DOI:** 10.3390/ijerph20054111

**Published:** 2023-02-25

**Authors:** Marco Antonio Hernández-Lepe, Melinna Ortiz-Ortiz, David Alfredo Hernández-Ontiveros, Minerva Janini Mejía-Rangel

**Affiliations:** 1 Medical and Psychology School, Autonomous University of Baja California, Tijuana 22390, Baja California, Mexico; 2 Sports School, Autonomous University of Baja California, Tijuana 22390, Baja California, Mexico

**Keywords:** elderly, inflammaging, cytokines, sarcopenia

## Abstract

Chronic, low-grade inflammation in the elderly, usually known as inflammaging, accelerates the development of age-related diseases, including cancer, obesity, sarcopenia, and cardio-metabolic diseases. Two of the most studied interventions against inflammation are diet supplementation and the regular practice of exercise. The search for this systematic review was performed in Scopus, EBSCO, and PubMed databases within the last 10 years. Only randomized controlled trials that evaluated the effect of supplementation and exercise against inflammatory markers in older adults were included. After applying eligibility criteria and risk-of-bias assessment, 11 studies were included in the systematic review. In total, 638 participants were analyzed and the main supplements evaluated were amino acid or protein supplementation from different sources. In the counterpart, the exercise applied in the evaluations included strengthening exercises or aerobic training. The interventions had a range of duration between 4 and 24 weeks, and the effects on inflammation markers in most of the studies showed a decrease in pro-inflammatory cytokines and non- or slightly significant change in anti-inflammatory cytokines. However, these results suggest that exercise and supplement interventions can contribute to diminishing the inflammation process in the elderly. We can also conclude that further well-designed randomized controlled trials are needed to confirm the possible synergistic effects of exercise and food supplementation against inflammation in the elderly due to the limited studies that currently exist. This systematic review was registered in PROSPERO, ID CRD42023387184.

## 1. Introduction

Inflammation is a physiological response of the body to restore homeostasis when any signal of tissue damage or pathogenic infection exists [1]. Chronic, low-grade inflammation plays a significant role in health and disease by accelerating the initiation and progression of age-related diseases, including cancer, obesity, sepsis, neuronal, and cardio-metabolic diseases [2]. In homeostasis, the organism has mechanisms to respond to any situation of physiological and pathological stress, resolving to stable levels in a short period of time until the body’s defensive function is completed. The phenotype of low concentration of inflammatory cytokines is usually observed in young people, and the presence of pro-inflammatory cytokines increases their concentration normally in situations of physiological or pathological stress. Conversely, when the body is unable to resolve the inflammatory process, the immune system maintains an activated phenotype and it may develop into chronic, low-grade inflammation [3].

In old age, persistent chronic inflammation is known as inflammaging [4]. Some cellular and molecular mechanisms related to inflammaging are: cellular senescence, mitochondrial dysfunction, abnormal mitophagy and autophagy, inflammasome activation, dysregulation of the ubiquitin–proteasome system, activation of DNA damage mechanisms, and dysbiosis [5]. Although the exact causes of inflammaging are unknown, a common cause involves the imbalance of the cytokine network and its homeostasis. Several molecular mechanisms are related with both aging and inflammaging, such as the molecular pathways that involve the transcription factor NF-κB and the pro-inflammatory cytokines IL-1, TNF-α, and IL-6, and the regulatory cytokine TGF-β that promotes an inflammatory phenotype [6]. The clinical consequences of inflammaging can be grievous, resulting mainly in an increased risk of developing diseases associated with metabolic syndrome, type 2 diabetes, cardiovascular disease, chronic kidney disease, cancer, depression, neurodegenerative diseases, rheumatoid arthritis, osteoporosis, and sarcopenia [7,8], reinforcing the urgent need to stablish treatments or interventions to reduce or avoid risk factors related to the development of these pathological conditions.

Sarcopenia is expressed as a progressive decline in strength, muscle mass, and physical function during aging, and is actually considered a health priority pathological condition in elderly research [9]. The most frequent complication associated with sarcopenia is less independence in the elderly in ordinary activities, as well as loss of strength in the extremities, physical disability, and poor quality life, resulting in negative health conditions, such as increased progression of chronic diseases, cognitive impairment, liver disease, disability, loss of independence, and death [10].

The most common strategies to prevent and treat sarcopenia are the promotion of regular exercise and the adequacy of a healthy diet/supplementation in the elderly. In this sense, there are many studies that carry out nutritional strategies or physical activity programs to prevent, maintain, or improve lean mass in older adults [11]. However, there are few studies dedicated to analyzing the inflammatory profile in older adults in response to a possible synergy between physical activity and diet supplementation. In this sense, the objective of the present review was to evaluate the effect of exercise together with dietary supplementation on the expression of inflammatory markers as a main outcome.

## 2. Materials and Methods

### 2.1. Study Search

This systematic review was carried out in accordance with the elements considered for planning a systematic review by Preferred Reporting Items for Systematic Reviews and Meta-Analyses (PRISMA) website [12], and the systematic review protocol was prospectively registered in the International Prospective Register of Systematic Reviews (PROSPERO), ID CRD42023387184.

Scopus, EBSCO, and PubMed databases were employed for this research. The search strategy was delimited by the following descriptors and Boolean operators: (“inflammation” AND (“Diet supplementation” OR “Supplement”) AND “Exercise” AND (“Elderly” OR “Older adults”)). Identified records were screened according to their publication date (within the last 10 years).

### 2.2. Elegibility Criteria

Articles analyzed in this review include full text of Randomized Clinical Trials (RCTs) that evaluate a synergistic effect of dietary supplementation and exercise or resistance training. Specific characteristics of RCTs were determined by PICO strategy, meaning:

- Participants: Older adults (age 60 and over) for both genders were included.
- Intervention/control groups: Studies that evaluated supplementation and/or exercise groups were included. Interventions of less than four weeks were excluded from the final selection.
- Comparison: Only RCTs where synergic treatment of supplementation and exercise groups were compared with control groups were included.
- Outcome measures: Only studies that reported evaluation of inflammatory markers as an outcome were selected.

### 2.3. Data Selection

Following the Cochrane Handbook for Systematic Reviews recommendations [13], two individual researchers (M.A.H.-L. and M.J.M.-R.) conducted the first database search, removed duplicate studies, and screened all titles and abstracts of the remaining data following the previous search strategy defined: limitations in the design (less than four weeks of duration as example) or implementation of the intervention, lack of control in the evaluations, inconsistency in the results, and the methodological quality of the selected studies (risk of bias) were assessed, screening the full texts and applying the Physiotherapy Evidence Database (PEDro) scale score [14]. In a scale range from 0 to 10 (0 or 1 value of each item), PEDro authors suggested that a methodological quality score of <4 is considered “poor”, 4 to 5 is considered “fair”, 6 to 8 is considered “good”, and 9 to 10 is considered “excellent”.

## 3. Results

### 3.1. Study Selection

**Figure 1:**
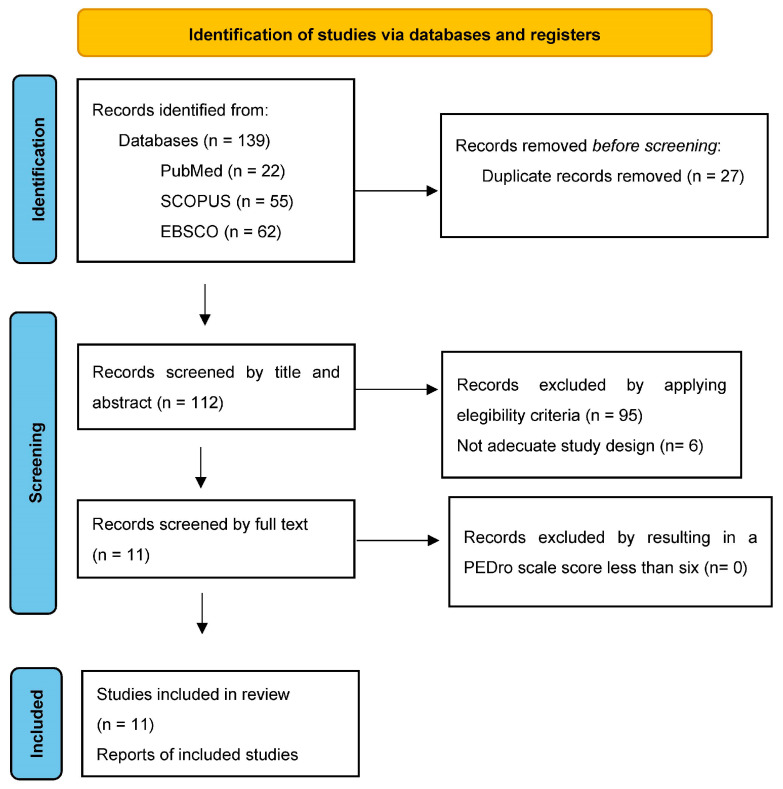
PRISMA 2020 flow diagram for new systematic reviews.

A flow diagram of the identification, screened, and included studies is shown in Figure 1. The initial search strategy showed 139 results, including the three aforementioned databases. After eliminating duplicates, all titles and abstracts were analyzed considering the eligibility criteria.

### 3.2. Methodological Quality/Risk-of-Bias Assessment

No studies were excluded for resulting in a PEDro scale score under six (Table 1). All eleven studies met the inclusion criteria and were over six in PEDro scale score and were included in this systematic review; however, two of them had the minimum score of six and one resulted in a total of seven in the scale score. 

### 3.3. General Characteristics of Included Studies

The included RCTs and their characteristics are described in Table 2. Participants in each study included ≥ 60-year-old adults; in total, 638 participants were analyzed together in the present review, and 55% of the studies evaluated amino acid or protein supplementation from different sources, with a particular bias towards specific amino acids, and one of those studies evaluated the influence of amino acids in conjunction with whey protein and vitamin D. A minority of studies (two) evaluated the effect of omega-3 supplementation compared with placebo. In the counterpart, exercise applied in the evaluations included strengthening, bodyweight resisted and resistance band exercises, balance exercises, or aerobic training. All the interventions had a minimum duration of four weeks, although the range of these was between 4 and 24 weeks. Further, a specification of the biological samples and technical approaches to each specific inflammatory marker of the studies are described in Table 3.

## 4. Discussion

The present systematic review was designed to evaluate the existing evidence of the effect of exercise together with food supplementation on inflammatory markers in older adults. Our results show that there is some scientific evidence regarding the anti-inflammatory effect of exercise or food supplementation alone [26,27]; a minimum number of randomized controlled trials have been carried out to study a possible additive or synergistic effect of both interventions.

It is well documented that physical activity promotes a strong inflammatory response, represented mainly by the recruitment of leukocytes and an elevated expression of circulating inflammatory mediators, secreted by immune cells and directly by the musculoskeletal tissue. Positive and negative effects on the immune system and susceptibility to mild illness symptoms have been observed in response to different physical activity protocols [4]. In the elderly population, the exercise interventions need to be feasible and safe for older patients. In this sense, the most common training protocols include resistance exercise, aerobic training, and combined resistance [28]. Most of the studies included in our review included resistance exercise [17,18,20,22], some consisted of combined training [15,21,24], and the last four consisted of different training approaches [16,19,23,25]. In a recent systematic review comparing the effects of exercise interventions on the inflammatory profile of older adults, researchers found that most consistent exercise effects on inflammation involved a reduction in circulating levels of CRP, IL-6, and TNF-α, which seemed more prominent in healthy older adults compared to those with a specific disease or condition [28]. These results are in agreement with ours, as we found that pro-inflammatory markers IL-6, TNF-α, and CRP decreased in six [15,16,17,19,20,23] of the eleven included studies. However, despite the multiple beneficial effects of physical activity, sometimes, negative physiological effects can occur when performing long-duration exercise sessions at vigorous intensity with a transitory function of the immune system, increasing inflammation and oxidative stress. To counteract the negative effects of vigorous exercise, reducing acute and chronic inflammation, and strengthening the immune response, healthy nutrition and food supplementation can be useful as intervention measures [29].

In this sense, handling of proteins, lipids, and carbohydrates in combination with certain food supplements could be a strategy to diminish the immune changes and inflammation associated with exercise [29]. Exercise may induce musculoskeletal and joint damage, increasing inflammatory cytokine levels. Some reports suggest that elevated concentrations of pro-inflammatory cytokines related to chronic inflammation promote protein degradation through the proteasome system and a reduction in musculoskeletal protein synthesis regulated by the protein kinase B/Akt downregulation pathway [30].

Proteins from soy and dairy products are rich in essential amino acids, show enough absorption kinetics, and include additional bioactive peptides that may confer nutritional benefits, in addition to those that stimulate musculoskeletal protein synthesis. Whey protein also has antioxidant properties due to its ability to improve the availability of the antioxidant enzyme system function and reduced glutathione. Soy protein and isoflavone-enriched soy protein can neutralize chronic inflammation through NF-κB pathway regulation and cytokine expression. Evidence suggests that protein from plant origins may be promising nutritional molecules against chronic inflammation and oxidative stress related to pathological conditions and inflammaging. Additionally, it has been demonstrated that the intake of moderate to high levels of omega-3 polyunsaturated fatty acids decreased the levels of pro-inflammatory cytokines in different populations [29], but information about the anabolic potential of dietary protein intake and food supplementation in older adults with elevated inflammaging is lacking [30]. Furthermore, micronutrients (vitamins C and D) can play an important role in immune function; in particular, they counterbalance the negative effects of oxidative damage due to free radicals. Some of these nutrients have potential anti-inflammatory properties, assessed by the attenuated levels of IL-6, TNF-α, and CRP [31]. As mentioned, there is a generalized effect of supplements enriched in proteins, amino acids, fatty acids, and vitamins on a decrease in pro-inflammatory cytokines; however, the action mechanisms differ between the different types of supplements.

Most of the studies included in the present review found a synergistic effect of physical exercise and diet supplementation on the expression of inflammatory markers. From the eleven studies included, seven analyzed circulating levels of inflammation markers (IL-6, IL-10, IL-1, CRP, TNF- α, IL-8) in serum or plasma samples [17,18,19,20,21,22,23,24] and one in saliva [15], and two studies included new markers, such as High-Mobility Group Box 1 (HMGB1) [19] and Monocyte Chemoattractant Protein-1 (MCP-1) [22].

Chronic, low-grade inflammation plays a central role in the progression of inflammaging and sarcopenia by modulating pro-inflammatory cytokines (TNF-α, IL-1, and IL-6. Cytokines TNF-α and IL-6), including ubiquitin–proteasome system pathway mediation by FOXO3a [32]. These cytokines may work synergistically to promote pathological conditions due to the cross-talk among immune cells and tissues, increasing protein degradation and reducing the protein synthesis, resulting in sarcopenia, functional impairment, and inflammaging [33]. HMGB1 is a nonhistone chromatin-associated protein involved in endocrine and metabolic anomalies related to systemic inflammation. HMGB1 increases cell proliferation and migration, stimulating the inflammatory status, becoming a pro-inflammatory cytokine [19]. High levels of HMGB1 are expressed in obesity and diabetes pathological conditions [34], and the protein is directly correlated with body mass index [35]. On the other hand, MCP-1 is a chemokine related with a state of chronic, low-grade inflammation [36], physical disability [37], sarcopenic obesity [38], and cardiovascular disease [39]. This is in response to oxidative stress, inflammation, or growth factors. MCP-1 acts as a chemotactic factor for monocytes, resulting in an adequate response to the inflammatory process [22]. Further, there is an increase in MCP-1 levels in senescent cells [40], which may suggest that high levels of MCP-1 play a role in aging [41,42].

Interestingly, four studies analyzed the intracellular levels of inflammatory markers at different levels of regulation and in specific tissues. Two studies used Peripheral Blood Mononuclear Cells (PBMCs) as a sample [24,25], one used adipose tissue [16], and the last one used skeletal muscle tissue [17]. Zychowska et al. [25] analyzed the gene expression of the cytokines IL-1, IL-6, IL-10, chemokine CCL2, and protein CRP in PBMCs. The authors report non-significant changes in the expression of classical markers of inflammation (IL-1, IL-6, IL-10, and CRP) but a significant decrease in CCL2 expression in the control group (placebo and physical activity).

Schober-Halper et al. [24] analyzed the gene expression of cytokine Transforming Growth Factor Beta, receptors 1 and 2 (TGF-β, TGF-β-RI, and TGF-β-RII), and the circulating levels of TGFβ and CRP protein. TGF-β is an anti-inflammatory and immunoregulatory cytokine involved in cell proliferation regulation, extracellular matrix production, inflammation, and immune functions [43]. Higher circulating levels of TGF-β are related to obesity with impaired insulin sensitivity [44], cardiovascular diseases [45], type II diabetes [46], and old age [47]. TGF-β interacts with several cell types by binding to membrane serine/threonine kinase receptors, TGF-βRI and TGF-βRII, initiating diverse cellular responses, depending on the ligand, the cellular context, and the stimuli [48]. In this study, the authors reported that TGF-β and TGF-βRII expression levels were non-significant in response to diet supplementation and physical activity; however, TGF-βRI expression levels decreased in the physical activity without diet supplementation group. Finally, circulating inflammatory markers were unaffected.

Cizkova et al. [16] used adipose tissue as sample and they found a direct correlation between the circulating levels of IL-6, IL-8, and TNF-α in conditioned medium from an adipose tissue culture compared with the levels in gene expression of IL-6, IL-8, and TNF-α from adipose tissue. In both cases, the levels of protein and mRNA of IL-8 and TNF-α decreased and a non-significant change in IL-6 was observed. Regardless of the complexity of this clinical trial, it had limitations, as it did not include a control or non-exercising group with or without omega-3 supplementation. The authors mentioned that the decision was based on both ethical and viability reasons. Finally, Dalle et al. [17] used skeletal muscle as a sample and reported that physical activity but no diet supplement (Omega-3) decreased inflammation (p65NF-κB) and catabolic (FOXO1) markers, and it improved muscle quality. This assumption is based on the inactivation of the transcription factors NF-κB and FOXO1 measured by the decrease in the phosphorylation of its activation residues Serine 536 and Threonine 24, respectively. However, non-significant changes in gene expression levels of IL-1β, TNF-α, and NFKB were observed. Circulating markers such as CRP remained unchanged between groups and IL-6 decreased in the supplement group.

Based on the intervention studies evaluated in this review, investigating the effects of physical activity and diet supplementation on inflammatory markers, such as CRP, TNF-α, IL-8, IL-10, and IL-6, the data are ambiguous because some studies revealed beneficial effects [15,16,23] while others did not observe any amelioration in the inflammatory state [16,17,20,21]. Others reported changes in only one kind of marker, pro- or anti-inflammatory [19,20,24,25]. This ambiguity in the effect of diet supplementation and physical activity on inflammation in the elderly may be related to many variables, such as: the duration of the intervention, the kind of diet supplement (protein, vitamins, antioxidants, fatty acids) and the dosage, the kind of physical activity, the study design, the number of participants, sex, age, healthy status, and the biological sample used for the analysis of inflammatory markers.

In this respect, recent studies are becoming more specific and are focusing on the analysis of inflammation markers in local and specific tissues (adipose, muscle) [16,17], specific immunological cell lineage [49], and also in organelles, such as mitochondria [50], probably to avoid the background of the systemic response of patients that, in most cases, can be related to the healthy status. They also use various techniques to determine the inflammatory markers, such as ELISA, RT-qPCR, transcriptional activity, and the activation of inflammatory pathways, that can include: nuclear factor kappa B (NF-κB), mammalian target of rapamycin (mTOR), and nuclear factor erythroid-related factor 2 (Nrf-2) signaling. These cell signaling pathways appears to play a central role in the pathophysiology of sarcopenia and frailty [51].

## 5. Conclusions

The present systematic review revealed that diet supplementation and physical activity may influence the inflammatory profile in older adults. However, positive, ambiguous, or null effects will depend on the specific diet supplementation, the type, time, intensity, and frequency of physical activity realized, and the design of the trial (use of control groups, duration, blinding). It is necessary that more controlled trials are performed to confirm the synergistic effect of physical activity and diet supplementation on inflammatory marker expression and regulation in the elderly population.

## Figures and Tables

**Table 1:** Physiotherapy Evidence Database of the screened studies by full text.

Study	Internal Validity Items								Statistical Reporting Items		Total Score
	RA	CA	BC	BP	BR	BAD	AFU	ITTA	BGSC	RPM	
[15]	1	1	0	0	0	0	1	1	1	1	6
[16]	1	1	1	1	1	0	1	1	1	1	9
[17]	1	1	1	1	1	1	1	1	1	1	10
[18]	1	1	1	1	1	1	1	1	1	1	10
[19]	1	1	1	0	0	0	1	1	1	1	7
[20]	1	1	1	1	1	1	1	1	1	1	10
[21]	1	1	1	0	0	1	1	1	1	1	8
[22]	1	1	1	1	1	0	1	1	1	1	9
[23]	1	1	1	1	1	1	1	1	1	1	10
[24]	1	1	1	0	0	0	0	1	1	1	6
[25]	1	1	1	1	1	1	1	1	1	1	8

RA: Random allocation; CA: Concealed allocation; BC: Baseline comparability; BP: Blinding of participants; BR: Blinding of researchers; BAD: Blinding of analyst of data; AFU: Adequate follow-up (>85%); ITTA: Intention-to-treat analysis; BGSC: Between-group statistical comparison; RPM: Reporting of point measures and measures of variability.

**Table 2:** Specific characteristics of the studies included in the systematic review.

Reference	Sample	Study and Duration	Supplement and Control	Exercise	Health Status	Inflammatory Markers Expression
[15]	83 (18 men and 65 women) 72.4 ± 6.1 years	Randomised controlled trial 4 weeks	L-glutamine 0.3 g/kg/day added to 10 g/day of maltodextrin Placebo (maltodextrin 10 g/day).	Combined-Exercise Training. Consisting of: Moderate aerobic exercises, aerobic training 30 min (postural stabilization and rhythm). Resistance exercise: 2–3 times a week, 5 to 10 different exercises (lower and upper limbs, abdomen, buttocks, and those related to postural stabilization, including dorsal and lumbar spine muscles).	Clinically stable	TNF-α (↓) and IL-10 (↑)
[16]	55 women 62–80 years	Cross-sectional and randomised interventional trial 16 weeks	Omega-3 and wax esters rich (5 capsules/day of calanus oil). The content of EPA + DHA in calanus supplementation was Approx. 0.23 g/day. Placebo (5 capsules/day of sunflower oil).	Physical fitness was evaluated by spiroergometry (maximum graded exercise test) and senior fitness tests.	Clinically stable	IL-6 (NS), IL-8 (↓), TNF-α (↓)
[17]	23 (8 women and 15 men) 65–84 years	Placebo-controlled, double-blind study and randomised 14 weeks	Omega-3 PUFAs softgels 1.1 g (1.02 g omega-3; 0.41 g DHA + 0.54 g EPA; 4 mg vitamin E) 3 times/week. Placebo (1.1 g corn oil in softgels).	Resistence exercise program 3 times per week for 12 weeks. Involved 3 sessions (~40 min) per week on nonconsecutive days.	Healthy	hsCRP (NS), IL-6 (↓) NF-κB (↓) TNF-α(NS), IL-1β (NS)
[18]	40 (15 men 59.1± 5.4 years; 25 women, 59.0 ± 6.7 years)	Double blinded 8 weeks	Colostrum 3 doses of 20 g/day (60 g/day total) Placebo (whey protein complex) containing about 38 g of protein per 60 g of complex.	Resistance exercise program (12 exercises, 3 sets of 8–12 reps, 3 days/ week).	Healthy	CRP (NS)
[19]	27 women, 67 ± 8 years	12 weeks	Vitamin D3 supplementation 800 or 4000 UI/day. Subjects were divided into two subgroups by different vitamin D baseline blood levels (LVD less than 20 ng/mL and MVD more than 20 ng/mL). However, supplementation varied across sub-groups.	1 h Nordic walking 3 times/week. Participants covered a total distance of 107 km 300 m.	Clinically stable	IL-6 (↓), HMGB1 (↓), IL-10 (NS)
[20]	36 men 67 ± 1 years	Randomised controlled, double-blind, 4-arm parallel group trial 12 weeks	Whey protein (25 g) + leucine (3 g) twice a day Placebo (23.75 g Maltodextrin).	Whole body resistence exercise, twice a week.	Healthy and active	IL-6 (↓), TNF-α (↓), CRP (NS) and IL-10 (NS)
[21]	100 (men and women) 68.73 ± 5.80 years	Randomised controlled trial 16 weeks	Leucine-enriched whey protein (0.5 g/kg/meal, 1.5 g/kg/day), based on body weight twice a day Control: No supplemented	Supervised and progressive exercise intervention comprising twice a week resistance exercise and once a week functional circuit exercises on non-consecutive days.	BMI: 27.06 ± 5.18 kg/m^2^	IL-6 (NS), TNF-α (NS), CRP (NS)
[22]	32 (men and women) 60–80 years	Pilot randomised, double-blind, placebo-controlled trial 12 weeks	Creatine 5 g/day Placebo (maltodextrin 5 g/day)	Resistance exercise Program 3 times/ week, 1 h of duration each session.	Healthy	IL-6 (NS), IL-10 (NS),CRP (NS), CCL2/MCP-1 (↓)
[23]	130 (77 women and 53 men) age ≥ 65 years	Randomised, controlled, double-blind, parallel-group superiority clinical trial 12 weeks	Vitamin D, essential aminoacids and whey protein, 32 g once a day at 1200 with meals Placebo (isocaloric amount of maltodextrin).	20 min exercise sessions, 5 times/week.	Sarcopenia	CRP (↓)
[24]	88 subjects age ≥ 65 years	Randomised controlled trial design with three parallel groups 24 weeks	Protein and vitamins consisted of 20.7 g protein (55 %; 19.7 g whey protein containing more than 10 g essential amino acids including 3 g leucine), 9.4 g carbohydrates (25%), 3.0 g fat (18%), 1.2 g fibre (2 %), vitamins such as vitamin D 800 IU (20 μg) , minerals and trace elements. 9 times a week.	Resistance exercise with elastic bands twice a week each session of 35–40 min.	Healthy	hsCRP (NS), TGF-β (NS), TGF-βRII (NS) TGF-βRI (↓)
[25]	24 women age ≥ 65 years	Randomised controlled trial 6 weeks	Vitamin C 1 g/day Placebo (cellulose in tablets).	Multidisciplinary exercise program 3 times/week for 1 h, consisted of gyrokinesis, Stabilization exercise, and Nordic walking at moderate intensity.	Healthy BMI under 25	IL-1 (NS), IL-6 (NS),IL-10 (NS),CRP (NS), CCL2/MCP-1 (↓)

BMI: Body Mass Index; CCL2/ MCP-1: C-C Motif Chemokine Ligand 2/ Monocyte Chemoattractant Protein-1; EPA: Eicosapentaenoic Acid; DHA: Docosahexaenoic Acid; CRP: C-reactive protein; hsCRP: high sensibility C-reactive protein; HMGB1: High Mobility Group Box1; IL-1: Interleukin-1; IL-6: Interleukin-6; IL-8: Interleukin-8; IL-10: Interleukin-10; NF-κB: Nuclear Factor Kappa B; PUFA: Polyunsaturated Fatty Acids; TGF-β: Transforming Growth Factor Beta; TGF-βRI: Transforming Growth Factor Beta Receptor 1; TGF-βRII: Transforming Growth Factor Beta Receptor 2; TNF-α: Tumoral Soluble Factor α; NS: Non Significance; (↓): Decreased concentration; (↑): Increased concentration.

**Table 3:** Experimental characteristics of the studies included in the systematic review.

Study	Biological Sample	Technical Approach	Inflammatory Markers
[15]	Saliva	ELISA	TNF-α, IL-10
[16]	Conditioned media and RNA from adipose tissue	Multiplex immunoassay and RT-qPCR	IL-6, IL-8, TNF-α
[17]	Serum and Skeletal muscle tissue	(1) ELISA (2) Western blot (3) RT-qPCR	(1) hsCRP (NS), IL-6 (2) p-NF-κB (3) TNF-α, N-κB, IL-1β
[18]	Serum	ELISA	CRP
[19]	Serum	ELISA	IL-6, HMGB1, IL-10
[20]	Plasma	ELISA	IL-6, TNF-α, CRP, IL-10
[21]	Serum	Multiplex immunoassay	IL-6, TNF-α, CRP
[22]	Plasma	(1) ELISA (2) Turbidimetric assay	(1) IL-6, IL-10, CCL2/MCP-1 (2) CRP
[23]	Serum	Nephelometric method	CRP
[24]	Plasma PBMCs	(1) ELISA (2) Cobas 8000 (3) RT-qPCR	(1) TGF-β (2) hsCRP (3) TGF-β,TGF-βRII, TGF-βRI
[25]	PBMCs	RT-qPCR	IL-1, IL-6, IL-10, CRP, CL2/MCP-1

CCL2/MCP-1:C-C Motif Chemokine Ligand 2/Monocyte Chemoattractant Protein-1; ELISA: Enzyme-Linked ImmunoSorbent Assay; CRP: C-reactive protein; hsCRP: high sensibility C-reactive protein; HMGB1: High Mobility Group Box1; IL-1 β: Interleukin-1 Beta; IL-6: Interleukin-6; IL-8: Interleukin-8; IL-10: Interleukin-10; mRNA: messenger RNA; NFκB: Nuclear Factor Kappa B; PBMCs: Peripheral Blood Mononuclear Cells; RT-qPCR: Reverse Transcriptase quantitative PCR; TGF-β: Transforming Growth Factor Beta; TGF-βRI: Transforming Growth Factor Beta Receptor 1; TGF-βRII: Transforming Growth Factor Beta Receptor 2; TNF-α: Tumoral Soluble Factor α.

## Data Availability

Data is available upon request to the first author (M.A.H.-L.).
